# Grandparents Raising Grandchildren: Applying Bioecological Theory to School Relationships and Grandparent Needs

**DOI:** 10.1093/geroni/igab046.3036

**Published:** 2021-12-17

**Authors:** Katharine Black, Nancy Mendoza

**Affiliations:** 1 The Ohio State University, Ottawa, Ohio, United States; 2 The Ohio State University, The Ohio State University, Ohio, United States

## Abstract

This paper examines the development, sustainability and importance of positive working relationships between grandparents raising grandchildren (“grandparents”) and school district staff through the application of Bronfenbrenner’s bioecological theory of human development. Grandparents have unique needs and often lack adequate resources, knowledge, and support when engaging in their new and often sudden role as primary caregiver of a grandchild. Access to educational resources, adequate information, and school district support is critical as a grandchild transitions into their grandparent’s home. This inquiry aims to develop a conceptual framework for understanding how forming and maintaining positive working relationships between grandparents and school district staff will systematically and adequately address the educational needs of grandparents and their grandchild’s academic success. Tenets of the bioecological model include the application of proximal processes that outline the need for frequent and regular interactions between a person and their environment over extended periods of time. The purpose of this study is to develop positive pathways of support through the application of the interconnected elements of proximal processes of the bioecological theory including process, person, context, and time, and the five bioecological interactive (micro-, meso-, exo-, macro-, chrono-) systems of human development. More specifically, educational needs of grandparents as caregivers are addressed through tenets of the bioecological theory to inform school districts and encourage the development of positive working relationships and effective education navigation protocols to better serve this unique and growing population.

